# Acknowledgement to Reviewers of *Journal of Fungi* in 2018

**DOI:** 10.3390/jof5010006

**Published:** 2019-01-09

**Authors:** Journal of Fungi Editorial Office

**Affiliations:** MDPI, St. Alban-Anlage 66, 4052 Basel, Switzerland

Rigorous peer-review is the corner-stone of high-quality academic publishing. The editorial team greatly appreciates the reviewers who contributed their knowledge and expertise to the journal’s editorial process over the past 12 months. In 2018, a total of 128 papers were published in the journal, with a median time to first decision of 16 days and a median time to publication of 33.5 days. The editors would like to express their sincere gratitude to the following reviewers for their cooperation and dedication in 2018:

Adenis, AntoineLeibundgut-Landmann, SalomeAimanianda, Vishu KumarLevesque, EricAkira, YoshimiLi, Shu ShunAlanio, AlexandreLiang, ZhihongAlessandrini, AuroraLiu, GangAlspaugh, J. AndrewLo, Hsiu-JungAmich, JorgeLopez-Llorca, Luis V.Ananda-Rajah, MichelleLowery, Thomas J.Andersen, BirgitteMadrid, HugoAnderson, MatthewMalacrinò, AntoninoBader, OliverMareș, MihaiBaker, ScottMartinez, LuisBarker, BridgetMatsuda, YudaiBates, StevenMcBride, Devin W.Bhatia, NealMcClelland, Erin E.Bicanic, TihanaMcCormack, Joseph G.Biernasiuk, AnnaMcculloch, MalcomBilitewski, UrsulaMcFeeters, RobertBillette, ChristopheMcNamara, LouiseBills, Gerald F.Mendoza-Mendoza, ArtemioBinder, UlrikeMeya, David B.Boekhout, TeunMiceli, Marisa H.Bogus, MieczyslawaMonk, BrianBongomin, FelixMorales-Cruz, AbrahamBorman, Andrew M.Moreira, José A. S.Bouklas, TejasMorio, FlorentBrannelly, LauraMorsy, MustafaBreak, TimothyMouyna, IsabelleBrown, Jessica C.Mucciarelli, MarcoBruno, VincentMunro, CarolBubici, GiovanniNaik, SurabhiBuil, Jochem B.Nakamura, TaroBuitrago, María JoséNanjappa, Som G.Cai, LeiNevez, GillesCalderone, RichardNiculita-Hirzel, HélèneCannon, RichardNkengfack, Augustin E.Carlson, RossNobbs, AngelaCaro, YanisNordøy, IngvildCasacuberta, ElenaNowacka-Jechalke, NataliaCassone, AntonioOehlers, StefanChang, Hao-XunOh, Seung-YoonChauhan, NeerajOlszewski, Michal AChen, Yee-ChunÖzenci, VolkanChlichlia, KaterinaPérez García, Luis AntonioChoi, Jong SooPerez, LaurentChung, KuangPeters, BrianCoelho, CarolinaPfaller, Michael A.Cogliati, MassimoPonikau, Jens U.Colombo, Arnaldo LopesPopolo, Laura MariaConti, HeatherPrado, SoizicConti, StefaniaQuindos, GuillermoCorzo-León, Dora E.Rajasingham, RadhaCosoveanu, AndreeaRamírez Soto, Max CarlosCrespo, Mariano SánchezRamonell, Katrina MariaCurrie, Amanda F.Ranque, StéphaneCytryńska, MałgorzataRath, Peter-MichaelDanesi, PatriziaRautemaa-Richardson, RiinaDannaoui, EricReed, Kevin F.M.Datta, KausikReynolds, HannahDavari, AgrinReynolds, Toddde Groot, Piet W. J.Richardson, JonathanDeng, PengRobert, VincentDesai, JigarRodrigues, Celia F.Diamond, GillRomo, JesusDrummond, Rebecca A.Sabino, RaquelEdgerton, MiraSánchez, Marta HerreraElad, DanielSarasan, ViswambharanElshahawi, SherifSavoie, Jean-MichelEnguita, Francisco J.Scher, Richard K.Erdman, ScottSchmidt, MartinEspinel-Ingroff, AnaSchütz, StefanEtxebeste, OierSchwarz, CarstenFaino, LuigiScorzoni, LilianaFalcó, VicençSegal, EstherFarnoud, AmirSerpico, RosarioFontaine, ThierrySeyedmousavi, AmirForche, AnjaShang, ZhuoFradin, ChantalSharp, JoFreimoser, FlorianSheehan, GerardFu, PengSheikh, AlaullahFuchs, Beth BurgwynShi, MeiqingGago, SaraSilar, PhilippeGarcia-Diaz, JuliaSimmonds, Daina H.García-Malinis, Ana JuliaSimões, Marta FilipaGarcia-Sherman, MelissaSimonetti, GiovannaGarnica, SigisfredoSingh, AnuragGelli, AngieSipsas, Nikolaos V.Giacobbe, Daniele RobertoSobrado, PabloGnavi, GiorgioSolano, FranciscoGonda, SándorSpakowicz, DanGong, LiminSpec, AndrejGóral, TomaszSpellberg, BradGreen, BenStadler, MarcGroll, AndreasStaniszewska, MonikaGuerrero, Javier PalmaStępień, ŁukaszHano, ChristopheSterflinger, KatjaHarper, JamesSturtevant, JoyHauser, Philippe M.Swidergall, MarcHayette, Marie-PierreT. Cornelison, ChristopherHe, FeiTafer, HakimHenao-Martinez, Andres FelipeTanaka, ShigeyukiHo, HonhingTaylor-Smith, LeanneHoffman, AngelaTesei, DonatellaHomma, TetsuyaTian, BinHoyer, LoisToffolatti, Silvia LauraIbrahim, AshrafTorren, Laura VilanovaIoannou, PetrosTreu, RolandJacobsen, IlseTullio, VivianJaiswal, Ashvin RUzman, DenizJeanmart, StephaneValiante, VitoJohnston, SimonVallini, GiovanniJoshi, SanketVan Ree, TeunisKalsi, MeghaVarese, Giovanna CristinaKataoka, RyotaVautier, SimonKauffman, CarolVíctor, González-MenéndezKavanagh, KevinVillani, SaraKellogg, JoshVinale, FrancescoKernien, John F.Vylkova, SlavenaKing, RobertWalther, GritKirkland, Theo N.Wang, ZheKirschner, RolandWatanabe, AkiraKlotz, Stephen A.Weidong, ChenKoide, Roger T.Wemheuer, FranziskaKolls, Jay K.Wilson, DuncanKornitzer, DanielWitherden, ElizabethKretschmer, MatthiasWong, Sarah Sze WahKrockenberger, MarkWoolford, CarolKucharikova, SonaXu, JintaoKumar, GauravXue, ChaoyangKumar, PradeepYaoita, YasunoriKumar, RohitashwYew, Wen ShanKumaresan, PappanaickenYurchenko, AntonLamoth, FrédéricZhang, RuiyongLau, AnnaZhao, YananLavergne, Rose-AnneZhou, BangjunLee, Chao-Hung


